# Paliperidone-Induced Massive Asymptomatic Creatine Kinase Elevation in Youth: From a Case Report to Literature Review

**DOI:** 10.3390/pediatric17010018

**Published:** 2025-02-07

**Authors:** Aurora Grandioso, Paola Tirelli, Gianmario Forcina, Vittoria Frattolillo, Delia De Biasio, Francesco Giustino Cesaro, Pierluigi Marzuillo, Emanuele Miraglia del Giudice, Anna Di Sessa

**Affiliations:** Department of Woman, Child, and General and Specialized Surgery, University of Campania “Luigi Vanvitelli”, 80138 Naples, Italy; grandiosoauro@gmail.com (A.G.); paolatirelli95@gmail.com (P.T.); gianmario.forcina@gmail.com (G.F.); vitto.fratt@gmail.com (V.F.); deliadebiasio@libero.it (D.D.B.); francescocesaro1993@gmail.com (F.G.C.); pierluigi.marzuillo@unicampania.it (P.M.); emanuele.miraglia@unicampania.it (E.M.d.G.)

**Keywords:** creatine kinase increase, asymptomatic, antipsychotic, paliperidone, youth

## Abstract

Background/Objectives: Unlike rhabdomyolysis and neuroleptic malignant syndrome (NMS), massive asymptomatic creatine kinase elevation (MACKE) represents a condition commonly detected during routine screening in patients receiving antipsychotic drugs. In particular, current evidence indicates a greater incidence of this condition in patients without signs of NMS, rhabdomyolysis, or other causes of CK increase during exposure to second-generation antipsychotics (SGAs) than first-generation antipsychotics (FGAs) with a variable onset and duration. Although its pathophysiology is still not fully elucidated, MACKE has usually been recognized as a self-limiting condition, but drug discontinuation might also be required to successfully revert it. Overall, knowledge in this field is mainly extrapolated from adult data, while similar evidence in youths is still limited. As clinicians might often deal with MACKE, its understanding needs to be expanded to avoid misdiagnosis, potentially leading to wasteful healthcare spending and unfavorable patient outcomes. Methods: By reporting the first case of MACKE in an adolescent receiving an SGA, namely paliperidone, we also aimed to provide a comprehensive overview of this medical condition. Conclusions: Making a MACKE diagnosis is essential since its relevant clinical and economic implications are mainly related to unnecessary closer laboratory monitoring or therapeutic changes (e.g., drug discontinuation or switch to another medication).

## 1. Introduction

In addition to the well-known role of trauma, viral infections, intramuscular injections, congenital myopathies, and intense physical activity as major causes of elevated serum creatine kinase (CK) levels in pediatric patients, certain medications such as antipsychotic drugs have also been implicated in this context both in adults and children [1,2,3,4,5]. Indeed, increased serum CK concentrations have been commonly reported in patients receiving antipsychotics, independently of drug class and dosage [1,4].

From a clinical point of view, this increase might be related to a wide spectrum of conditions ranging from neuroleptic malignant syndrome (NMS) and rhabdomyolysis to massive asymptomatic creatine kinase elevation (MACKE) [4,5].

While NMS and rhabdomyolysis are well-characterized medical disorders [1,3,4], MACKE represents an autonomous clinical entity (or possible side effect) with CK elevation during antipsychotic exposure in the absence of any additional NMS sign or other well-known causes of CK elevation (e.g., rhabdomyolysis, myocardial infarction, thyroid dysfunction, muscle injuries or syndromes, trauma, exercise, etc.) [2,5]. It might occur during the first days of treatment or several months (up to two years) after starting antipsychotics, with a variable duration from a few days to four weeks [2,5]. However, MACKE generally represents a self-limiting or reversible condition after drug discontinuation, but its exact pathophysiology is still unclear [2,4,5].

Several case reports described MACKE in adult psychotic patients, particularly in those receiving second-generation antipsychotics (SGAs) (e.g., olanzapine, risperidone, quetiapine, etc.) and, more rarely, first-generation antipsychotics (FGAs) (e.g., haloperidol, loxapine, molindone thioridazine, and perphenazine) [2,3,4,6,7,8]. Overall, the use of SGAs instead of FGAs in patients with psychotic spectrum disorders has been found to have a more positive impact on their quality of life, likely due to their better tolerability profile [9].

However, evidence regarding MACKE in children and adolescents receiving antipsychotics is still scarce [5,10,11]. Given also the potential clinical and economic implications related to harmful overdiagnosis [2,5], a deeper knowledge of MACKE is needed.

Starting from a clinical case, we aimed to provide a more comprehensive overview of MACKE during antipsychotic treatment in pediatric patients. Informed consent to publish the results was obtained from the patient and his parents.

## 2. Case Report

A 16-year-old boy was followed up in our Nephrology Clinic for chronic kidney disease (CKD) secondary to severe left vesicoureteral reflux. His medical history also included obesity, which was managed through diet and lifestyle changes. Due to CKD-related proteinuria, he was also treated with angiotensin-converting enzyme inhibitors (ACE) and angiotensin II receptor blockers (ARBs), and he showed a good clinical response to this regimen.

The patient developed a neuropsychiatric disorder characterized by aggression, mood swings, and emotional instability during the first COVID-19 lockdown. Symptoms included irritability, heightened frustration, and impulsive outbursts, often triggered by minor events. Additionally, the patient experienced mood fluctuations that contributed to overall emotional dysregulation, significantly impacting daily functioning and behavior. Therefore, he was referred to a neuropsychiatrist and he was initially prescribed an oral starting dose of paliperidone of 3 mg/day, which was gradually adjusted based on clinical response and tolerability. After titration, the patient stabilized on a maintenance dose of 7.5 mg/day for a duration of 12 months. Throughout this period, the medication effectively addressed the psychiatric symptoms and was well tolerated, with no clinical or laboratory abnormalities observed.

However, after 12 months of treatment, a significant increase in CK levels was detected during a routine blood test. Notably, this elevation occurred without any other abnormalities or clinical symptoms.

Indeed, during the last nephrology follow-up visit, the general physical examination was unremarkable, and the psychiatric symptoms had improved. Routine blood tests revealed an elevated CK serum level of 2436 U/L, which rapidly increased to a peak of 7489 U/L within two days. Despite this significant rise, the patient remained asymptomatic. Further evaluations, including cardiac, hepatic, muscle, and neurological assessments, along with serum troponin, myoglobin, CK isoforms, urinalysis, myoglobinuria, and uricosuria, were all within normal limits. Serum creatinine levels were stable and comparable to those observed in previous follow-up visits. No evidence of endocrinological, infectious, or electrolyte disturbances that could explain the CK increase was found. Genetic variations in drug-metabolizing enzymes, such as CYP2D6 and CYP3A4, were not ruled out.

In response, paliperidone was discontinued, and the patient received intravenous hydration. Both ACE and ARBs were continued, as they are not commonly linked to significant CK elevation

A follow-up after 96 h showed a decrease in CK levels, which dropped to 564 U/L, with no clinical symptoms present. After a diagnosis of MACKE was made, and following consultation with the neuropsychiatrist, the decision to discontinue paliperidone was confirmed.

A subsequent laboratory evaluation one week later showed normalization of CK levels, and the patient continued to exhibit stable and positive psychiatric behavior. Given the good control of psychiatric symptoms and the progressive normalization of CK levels, no alternative treatments were considered after paliperidone discontinuation.

Paliperidone was gradually reintroduced, increasing up to 7.5 mg/day, with further improvement in psychiatric symptoms and no laboratory abnormalities. After three-month follow-up, CK levels decreased further to 227 U/L and the psychiatric picture of the patient further improved. At the last clinical encounter, the patient remained on paliperidone with normal CK levels and well-controlled psychiatric symptoms.

## 3. CK Elevation and Antipsychotic Drugs: Potential Pathophysiological Mechanisms

Various factors have been implied in CK increase associated with antipsychotics, particularly paliperidone, a dopamine D2 receptor antagonist [5,12,13].

These include extrapyramidal symptoms (EPS), which cause—through dopaminergic dysregulation—muscle rigidity and strain, and NMS, leading to muscle breakdown. Additionally, dopamine receptor blockade by paliperidone can disrupt normal muscle function and coordination, contributing to rigidity, dystonia, and muscle injury. EPS, such as tremors and bradykinesia, can place additional strain on muscles, further promoting CK release [5,12,13].

Certain external factors such as lifestyle and environmental stressors, such as intense physical activity, intramuscular injections, the use of restraints, dehydration, electrolyte imbalances, poor nutrition, dystonic reactions, heat exposure, myopathies, infections, and overdoses of various substances (e.g., alcohol, cocaine, or amphetamines), when combined with the effects of antipsychotic medications, might also potentially contribute to CK increase [3,4].

Although its exact pathophysiology is not yet fully understood [5,11], a state-dependent vulnerability or sustained by unidentified exogenous factors has been suggested [5,12,13].

**Figure 1:**
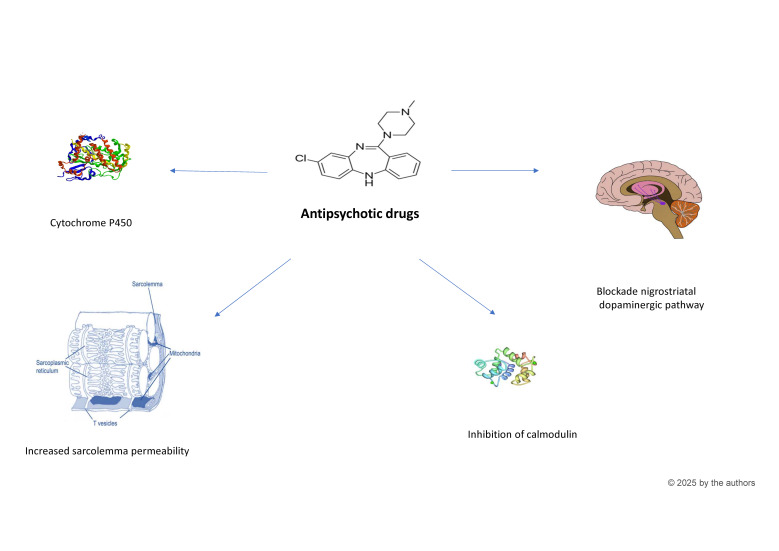
Potential pathophysiological mechanisms of MACKE during antipsychotic treatment.

In particular, evidence suggested the involvement of multiple non-alternative pathways in MACKE development [3,5,12,13] (Figure 1).

Among the proposed molecular mechanisms, the increase in sarcolemma membrane permeability, which allows CK to leak from muscle cells into the plasma, has been widely studied and is currently considered the most reliable hypothesis [5,12,13,14,15]. This phenomenon may result from serotonin receptor antagonism, which impairs glucose uptake [5,12,16,17]. Specifically, the binding of certain antipsychotics to high-affinity 5-hydroxytryptamine (5-HT)2 receptors in the sarcolemma can lead to an accumulation of 5-HT through passive diffusion. This, in turn, increases membrane permeability and results in higher serum CK levels [5,13]. Another potential mechanism involves the inhibition of calmodulin or protein kinase C function, which could lead to CK leakage from muscle cells [3,5,17].

Additionally, a direct effect of psychotropic drugs on CK synthesis within muscle cells has been suggested as a further, though less studied, pathogenic link [3,5,11,17]. The blockade of the nigrostriatal dopaminergic pathway is another potential central mechanism that could contribute to CK elevation [3,5,12,13]. This pathway has been implicated in involuntary movements, rigidity, stiffness, and akathisia, all of which could lead to increased CK levels [3,5,12,13]. However, evidence supporting this hypothesis is mixed, as the higher incidence of rhabdomyolysis reported in patients treated with quetiapine—known for its relatively lower D2 receptor blockade—raises questions [14].

Given its role in the metabolism and elimination of some psychotropic medications [5,9,18], the contribution of cytochrome P450 enzymes to CK elevation should also be considered [5,12,13]. Although the evidence remains limited, genetic variations in drug-metabolizing enzymes, such as CYP2D6 and CYP3A4, can affect the pharmacokinetics of the drug, explaining the variability in CK increases during exposure to certain antipsychotics [12,16].

Moreover, metabolic changes induced by antipsychotics may also interfere with muscle repair mechanisms, increase oxidative stress, and disrupt energy production, all of which can contribute to muscle damage and elevated CK levels [3,12,13].

Furthermore, paliperidone may cause electrolyte imbalances and dehydration, which can heighten the risk of muscle injury. Its sedative effects may reduce physical activity, leading to increased muscle stiffness [3,11,12].

Taken together, these mechanisms underscore the complex interaction between drug effects on the central nervous system and peripheral muscle function [3,5,12].

However, most of the existing evidence comes from adult studies [3,19,20,21,22], and the effects in younger patients may differ. As a result, clear evidence on the mechanisms of CK elevation in youth, particularly with paliperidone, remains limited.

Despite these intriguing hypotheses, further research is needed to fully understand the pathophysiology of MACKE during antipsychotics treatment.

## 4. CK Increase and Antipsychotics Treatment: From Adult to Childhood Evidence

Evidence on the association between CK elevation and antipsychotic drugs is mainly supported by case reports in adult patients [3,19,20,21,22], while pediatric data are scarce [5,9,11,17] (Table 1).

In line with the findings in adults [1,2,3,5], CK elevation has been predominantly reported in adolescents treated with SGAs [3,15,20,21,22,23], rather than FGAs [7]. This increase typically occurs between 2–3 weeks and 18 months after starting therapy [2,5,6,7,8].

While robust data have demonstrated the association of both neuroleptic malignant syndrome (NMS) and rhabdomyolysis with antipsychotic treatment in adults [2,7,23,24,25], some evidence suggests that MACKE can occur regardless of the drug type, regimen, or therapeutic scheme (e.g., monotherapy or polytherapy) [2,5]. Although MACKE is usually a self-limiting condition, in some cases, CK levels normalize after drug discontinuation [2,5,10]. Notably, rechallenge with the same antipsychotic or switching to another medication has been linked to the recurrence of MACKE, supporting the hypothesis of a state-dependent vulnerability [5,12,16].

However, case reports describing similar associations in pediatric populations are sparse [5,9,11,17]. Consistent with adult findings [2,7], elevated CK levels have primarily been observed in adolescents aged 11–19 years treated with SGAs [5,10,11,17]. The onset of the condition ranged from 2 to 3 weeks to 18 months after starting therapy [5,10,11,17].

A notable case involved a 16-year-old boy who developed a CK peak of 10,350 U/L 18 months after starting olanzapine, without signs or symptoms of NMS, as described by Masi et al. [5]. Upon discontinuation of olanzapine, CK levels gradually decreased over the course of a week and normalized within two weeks [5]. However, a slight elevation in CK, along with mild increases in aldolase and creatinuria, appeared about two months after reintroducing the therapy. Despite normal cardiac, neurometabolic, and muscle evaluations, olanzapine was reinitiated with a favorable clinical response and complete laboratory normalization [5]. These authors also described two cases of significant CK elevation (3300 U/L and 7284 U/L) in two youths treated with risperidone [5]. Management varied between cases: one patient had risperidone discontinued, resulting in CK normalization a few weeks later, while the other patient, despite a higher CK peak, continued the medication due to an improvement in psychiatric symptoms and the absence of clinical impairment. In this case, CK levels decreased significantly (below 500 U/L) within two weeks [5].

In a similar case, a 17-year-old boy treated with risperidone developed a CK peak > 9000 U/L three weeks after starting therapy [10]. CK levels normalized after one week following discontinuation of the medication [10].

In addition to drug discontinuation, a “wait-and-see” strategy has also been effective in normalizing CK levels without symptoms, with concurrent improvement in psychiatric features in young patients receiving risperidone, quetiapine, aripiprazole, and clozapine [2,7,17]. Similarly, a patient aged 11 years on quetiapine presenting with CK increase demonstrated CK normalization within 8 days after continuing the medication. A later CK elevation occurred after adding aripiprazole and clozapine, but following the same strategy, CK levels gradually decreased and returned to normal by day 114 [11].

## 5. MACKE During Antipsychotics: A Practical Management

Overall, the potential for MACKE during antipsychotic treatment should be recognized. Importantly, its occurrence does not appear to be influenced by the specific antipsychotic or its dosage [5,11]. Timely and careful clinical monitoring, including regular assessments of liver, heart, and kidney function, is essential [3,11,17].

However, based on the limited available evidence (mainly resulting from case series) [5,26,27,28], immediate discontinuation or switching of the antipsychotic is typically unnecessary [3,5,11]. No antipsychotic can be considered entirely risk-free, and there is currently no evidence supporting the necessity of switching to another medication, even one with a different receptor profile [3,5,11].

In cases where CK levels are below 3000 U/L and no severe clinical symptoms are present, a “wait-and-see” approach is generally advisable, as CK levels often begin to decrease within a few days [3,5,11,17]. However, close monitoring is crucial to detect early signs of complications, such as NMS or rhabdomyolysis. Warning signs may include fever, muscle rigidity, altered consciousness, signs of kidney damage, and autonomic instability (e.g., fluctuations in body temperature, electrolytes, blood pressure, or heart rate) [3,5].

If CK levels exceed 5000 U/L and persist for more than two weeks, reducing the dose or discontinuing the antipsychotic is recommended. If CK levels normalize and the psychiatric condition worsens significantly, reintroducing the same medication may be considered, although the outcomes of this approach remain unpredictable [5].

However, given the lack of robust pediatric evidence in the field [29], there are no current clear guidelines for MACKE management in youths receiving antipsychotics.

## 6. Conclusions

Given the increasing use of antipsychotic medications in younger populations [30,31], clinicians may frequently encounter cases of CK elevation [2,4,5]. However, while robust evidence exists regarding MACKE in adult patients receiving antipsychotics [2,7,23,24,25], similar data for pediatric populations remain limited [5,10,11,17]. This review, beginning with the first reported case of MACKE in a young patient treated with paliperidone, expands and supports current pediatric knowledge in this area. Paliperidone, a well-known SGA, presents not only with a good tolerability profile [9], but also with positive effects on non-core symptoms [32], globally improving the quality of life of patients with psychotic spectrum disorders [9].

Since MACKE shows distinct features compared to NMS and rhabdomyolysis, accurate differential diagnosis is essential. Misdiagnosis could lead to unnecessary interventions, resulting in wasted healthcare resources and poor patient outcomes [2,4,5]. Timely diagnosis of MACKE can help to avoid unnecessary close laboratory monitoring and the premature discontinuation or switching of effective medications. In cases with high (>5000 U/L) and persistent (for more than two weeks) CK levels, dose reduction or discontinuation may be recommended. In asymptomatic patients with moderate CK elevation and no signs of NMS or rhabdomyolysis, a “wait-and-see” strategy may be appropriate [2,5].

Increased awareness of MACKE could facilitate a deeper understanding of its pathophysiology and epidemiology, leading to improved management strategies for these young patients.

## Figures and Tables

**Table 1:** Main pediatric evidence of a CK increase during antipsychotic treatment.

Author [Ref.]	Year	Antipsychotic Drug	Drug Receptor Profile	Dose (mg/day)	Sex	Age (Years)	CK Increase Onset	CK Peak (UI/L)	Discontinuation (yes/no)	Normalizationof CK
Masi et al. [5]	2014	Risperidone	5HT2A, D2, α1, H1	3	M	13	1 year	3300	Yes	Yes, in few weeks
		Risperidone		1.25	M	14	2 months	7284	No	No (reduction yes)
		Olanzapine	5HT2A, D2, α1, H, 5HT2C, M1 antagonism	10	M	16	18 months	10,350	Yes	Yes, in 2 weeks
Holtmann et al. [10]	2003	Risperidone		3.5	M	17	3 weeks	9743	Yes	Yes, in 1 week
Bachmann et al. [11]	2007	Quetiapine	5HT2A, D2, α1, H1, M1	1200	M	11	4 weeks	1088	No	Yes, in 8 days
		Aripiprazole	D2 antagonism (in mesolimbic system) Partial D2 agonism (mesocortical system)	10	M	11	14 weeks	4572	No	Yes, in 8 days
Boot et al. [17]	2000	Olanzapine		20	M	19	6 weeks	6840	No, dose reduction	No (reduction yes)
		Quetiapine	Quetiapine	NR	M	19	NR	3942	No	No (reduction yes)

Abbreviations: M, male; NR, not reported; 5-HT: 5-hydroxytryptamine.

## Data Availability

The original contributions presented in the paper are included in the article, further inquiries can be directed to the corresponding author.
